# Bankart repair and remplissage for surgical treatment of first episode of acute anterior shoulder dislocation: long-term results

**DOI:** 10.1007/s00590-025-04496-x

**Published:** 2025-08-30

**Authors:** Pascal Malandri Ghipponi, Bahu Edouard, Marc Limousin, Guillaume Strouk, Pierre-Emmanuel Ridon, Franck Remy, Eric Meyer, Aurélien Moulin-Traffort, Thomas Amouyel, Carlos Maynou, Clement Dambielle

**Affiliations:** 1 Centre Hospitalier Universitaire de Lille, Lille, France; 2 Clinique Chirurgicale de Saint-Omer, Saint-Omer, France

**Keywords:** Anterior shoulder instability, Bankart repair, Remplissage procedure, Recurrence and return to sport

## Abstract

**Background:**

Shoulder instability is a common condition among young athletes, with high recurrence rates following conservative treatment. Bankart repair is a widely used surgical approach, but its failure rate remains significant, even for non-engaging defects. Combining Bankart repair with remplissage has been proposed to reduce recurrence rates.

**Methods:**

This retrospective, single-center study analyzed the long-term outcomes of young athletes (18–30 years old) who underwent Bankart repair combined with remplissage after a first anterior glenohumeral dislocation. Patients were evaluated at least five years postoperatively for recurrence rates, functional outcomes (Walch-Duplay, WOSI, Quick-DASH), return to sports, and the need for revision surgery.

**Results:**

Twenty-five patients were included, with an average follow-up of 81.4 months. The recurrence rate was 12% (3/25), with one patient requiring revision surgery. Functional scores were satisfactory (Walch-Duplay: 89.5, WOSI: 10.95, Quick-DASH: 14.30), and 84% of patients returned to sports at their previous level or higher. No significant complications were reported.

**Conclusion:**

Bankart repair combined with remplissage appears to be an effective first-line treatment for young athletes with a first anterior shoulder dislocation, achieving a low recurrence rate and high return-to-sport levels. Further prospective, comparative studies are needed to validate these findings.

## Introduction

Shoulder instability is a common condition, particularly among young athletes. The recurrence rate of dislocation following orthopedic treatment is high, reaching up to 90% in certain studies [1–3]. Several studies recommend immediate Bankart surgery for young athletes [4–11], as it provides significantly better outcomes than conservative treatment and is associated with a low complication rate. While this approach has shown promising advances in the management of these patients, the failure rate of isolated Bankart repair remains high in most publications, ranging from 22 to 31% in the long term [12, 13]. This failure rate could partially be attributed to the presence of a postdislocation defect, which may predispose the patient to chronic instability. Several studies have explored whether these defects are engaging or not [14]. It has been observed that the failure rate of Bankart surgery remains elevated even in cases where the defects are considered non-engaging. Recent studies have shown that when Bankart repair is combined with a remplissage procedure, recurrence rates of dislocations are lower than those associated with isolated Bankart repairs, even when the defect is classified as non-engaging [15, 16] potentially offering a less invasive alternative to bone-block procedures such as the Latarjet. While the Latarjet technique is widely accepted for recurrent dislocations or off-track lesions, it is associated with higher surgical complexity and potential complications, including graft non-union, neurovascular injury, and limitations in external rotation [17]. Bankart repair with remplissage may provide a middle ground by enhancing stability while preserving native anatomy and minimizing risks.

The aim of our study is to present the long-term results of surgical stabilization using antéro-inferior labrum repair combined with remplissage for the management of first-time anterior shoulder dislocations in young athletes under 30 years of age, regardless of whether the defect is considered engaging or non-engaging.

## Materials and methods

This is a retrospective, single-center study conducted at the Saint Omer Surgical Clinic. Patients were included from March 2014 to March 2016. Follow-up occurred at least 5 years after their first dislocation episode to collect outcome measures as described in Sect. “Discussion” of the Materials and Methods, either through in-person consultations or teleconsultations.

All patients who underwent surgery had preoperative arthro-CT scans to assess the engaging (off-track) or non-engaging (on-track) nature of the humeral defect, according to the technique described by Di Giacomo [18]. Table 1 summarizes the characteristics of the included patients.

**Table 1:** Characteristics of study groups

Sex (male/female): N (%)	19 (76%)/6 (24%)
Age at inclusion (years): median (IQR)	21.5 (20–22.5)
Dominant side (right/left): N (%)	21 (84%)/4 (16%)
Height (cm): median (IQR)	175 (169–180)
Weight (kg): median (IQR)	79 (64–88)
BMI (kg/m^2^): median (IQR)	23.1 (20.9–26)
Affected side (right/left): N (%)	16 (64%)/9 (36%)
Associated injuries	0
Professional activity: N (%)	11 (44%)
Competitive-level sports N (%)	8 (32%)
Collisions athletes N (%)	9 (36%)
Off-track lesion N (%)	6 (24%)
Mean time to surgery (weeks)	6,9 (3–11)
Total number of patients	25

IQR: interquartile range; BMI: body mass index

A total of 25 patients were included in the study. The cohort consisted predominantly of males (76%), with a median age at inclusion of 21.5 years (interquartile range [IQR]: 20–22.5). The dominant arm was affected in 84% of cases, and the right shoulder was involved in 64% of patients. The median height was 175 cm (IQR: 169–180), with a median weight of 79 kg (IQR: 64–88) and a median body mass index (BMI) of 23.1 kg/m^2^ (IQR: 20.9–26). No associated injuries were noted at baseline.

Regarding activity level, 44% of the patients had ongoing professional activity, 32% participated in competitive-level sports, and 36% were involved in contact sports. Preoperative imaging identified off-track lesions in 24% of patients. The mean time from initial dislocation to surgical intervention was 6.9 weeks.

### Inclusion criteria

Patients aged 18–30 who underwent Bankart repair combined with remplissage for the management of a confirmed first episode of anterior glenohumeral dislocation (LGHA) on radiographs were included. Additionally, all patients were required to practice a sport.

### Exclusion criteria

Exclusion criteria included:

- Non-traumatic dislocation with hyperlaxity, defined as a Beighton score ≥ 4.
- Associated humeral head fractures.
- Pregnancy or lactation.
- Patients under legal guardianship or those unable to provide informed consent.
- Recurrent dislocation

### Study procedure

Patients were re-contacted at least 5 years postsurgery. They were offered a physical consultation, teleconsultation, or a phone interview to gather the following data:

- Recurrence of dislocation requiring reduction by a third party and the frequency of such occurrences.
- Episodes of subluxation characterized by acute painful episodes with sensations of catching, dislocation, or locking that spontaneously resolved following self-reduction maneuvers.
- The need for secondary surgical intervention in patients who have undergone surgery
- Return to sport, specifying whether the return was at the same or lower level.
- Functional scores: WOSI [19], DASH [20], and Walch-Duplay [21] (completed and returned by email or mail for patients not seen physically).

### Outcome measures

The primary outcome measure was the occurrence of recurrent glenohumeral instability.

Secondary outcome measures included:

- Sensation of instability.
- The need for secondary surgical intervention after initial inclusion.
- Functional scores

### Surgical technique

The procedure was performed under general anesthesia (GA) in a semi-sitting position using an arthroscopic technique by a shoulder-specialized surgeon, following the method described by Wolf [22]. A posterior viewing portal and an anterior working portal through the rotator interval were established. After diagnostic arthroscopy and confirmation of a Bankart lesion and Hill-Sachs defect, the Hill-Sachs lesion was prepared using a burr to promote bleeding bone. Two non-absorbable suture anchors were inserted percutaneously into the defect, and sutures were passed through the infraspinatus tendon and posterior capsule using a suture-passing device. Knots were tied in the subacromial space to complete the remplissage tenodesis. The scope was then reintroduced into the joint, and the capsulolabral complex was mobilized and prepared. Depending on lesion size, two or three suture anchors were placed along the anteroinferior glenoid, and the labrum and capsule were repaired using non-absorbable sutures in simple or mattress configuration. Portals were closed and a standardized postoperative rehabilitation protocol was initiated.

### Statistical analysis

Continuous variables were expressed as means, standard deviations (SD), and ranges (minimum–maximum). Categorical variables were described using frequencies and percentages. Normality of data distribution was assessed using the Shapiro–Wilk test. Comparisons between groups (e.g., recurrence vs. non-recurrence, return to sport at same level vs. lower level) were made using the Student’s t test (parametric data) or Mann–Whitney U test (nonparametric data) for continuous variables. For categorical variables, comparisons were made using the Chi-square test or Fisher’s exact test when appropriate. A significance level of *p* < 0.05 was considered statistically significant. Statistical analyses were conducted using R software, version 3.6.1 (R Foundation for Statistical Computing, Vienna, Austria).

This study was conducted in accordance with the ethical principles outlined in the Declaration of Helsinki and was approved by our institutional Ethics Committee (Clinical Research Federation). All included patients were informed about the objectives and procedures of the study, and oral informed consent was obtained prior to their inclusion. Participants’ personal data confidentiality was strictly maintained throughout the study in compliance with the European General Data Protection Regulation (GDPR).

## Results

A total of 25 patients were successfully re-evaluated, with the majority (88%, 22/25) opting for teleconsultations due to geographic constraints.

Three patients (12%) experienced recurrent instability postoperatively. All three cases involved recurrent glenohumeral dislocation requiring reduction by a third party, and two of these patients also reported subluxation episodes characterized by acute pain and a sensation of catching or shifting. Among them, one patient had an off-track lesion preoperatively, which may have contributed to the recurrence. Recurrences occurred between the third and fifth postoperative years, with one patient ultimately requiring revision surgery with a bone-block procedure after multiple dislocations. The main findings are shown in Table 2.

**Table 2:** Main findings of the study

Recurrent anterior dislocation N (%)	3 (12%)
Recurrent anterior dislocation with off track lésion N (%)	1 (4%)
Presence of subluxation N (%)	2 (8%)
Return to sport at the same or higher level N (%)	21 (84%)
Need for secondary surgical stabilization	1 (4%)

When stratified by preoperative humeral head defect type, 19 patients (76%) had on-track lesions and 6 patients (24%) had off-track lesions. Among the three recurrences observed, two occurred in the on-track group (10.5%) and one in the off-track group (16.7%). The off-track recurrence required revision surgery with a bone-block procedure. The detailed distribution of recurrences by lesion type is presented in Table 3.

**Table 3:** Recurrence according to preoperative on-track/off-track status

Lesion type	n (%)	Recurrences n (%)	Need for revision n (%)
On-track	19 (76)	2 (10.5)	0 (0)
Off-track	6 (24)	1 (16.7)	1 (16.7)
Total	25 (100)	3 (12.0)	1 (4.0)

Postoperative functional outcomes were generally favorable. The mean Walch-Duplay score was 89.5 (range: 50–100), reflecting excellent shoulder function. The mean WOSI score was 10.95 (range: 0–51), indicating minimal shoulder-related disability. The mean Quick-DASH score was 14.30 (range: 11–31), suggesting good overall upper-limb function.

All patients successfully returned to sports, with 84% (21/25) resuming their activity at the same or a higher level than before the injury. The remaining 16% (4/25) returned to sports at a slightly lower intensity, primarily due to personal preference rather than functional limitations. No patients reported a complete cessation of sports participation due to persistent shoulder instability.

No major complications were observed during the postoperative period. The average scores based on the group are shown in Table 4.

**Table 4:** Functional scores (Walch-Duplay, Quick-Dash, and WOSI)

Bankart + Remplissage	Mean ± SD (Min–Max)
Walch-Duplay	89.5 ± 16.05 (50–100)
A (Activities of daily living)	23.91 ± 4.22 (10–25)
B (Stability)	19.75 ± 6.78 (0–25)
C (Pain)	22.25 ± 4.99 (10–25)
D (Global mobility)	23.25 ± 4.38 (10–25)
WOSI	10.95 ± 15.61 (0–51)
Quick-Dash	14.30 ± 6.33 (11–31)

SD: standard deviation; Min: minimum; Max: maximum

A multivariate logistic regression analysis was performed to identify independent qualitative factors associated with shoulder dislocation. Three factors were analyzed: **competitive sport level** (β = 0.51, *p* = 0.708), **participation in contact sport** (β = 0.36, *p* = 0.793), and the presence of an **Off-track lesion** (β = 0.00, *p* = 0.528). None of these variables independently demonstrated a statistically significant association with shoulder dislocation (all *p* values > 0.05). Results are shown in Table 5.

**Table 5:** Factors associated with shoulder dislocation (multivariate logistic regression)

Factor	Coefficient (β)	*p* value
Competitive sport level	0.51	0.71
Contact sport	0.36	0.79
Off-track lesion	0.10	0.53

## Discussion

The results of this study demonstrate that arthroscopic Bankart repair combined with remplissage provides favorable long-term outcomes in young athletes following a first-time anterior shoulder dislocation. With a recurrence rate of only 12% after an average follow-up of 81.4 months, this combined technique offers significant stability while maintaining excellent functional scores and a high rate of return to sport. These findings are particularly relevant given that the failure rate of isolated Bankart repairs in the literature remains high, ranging from 22 to 31% [12, 13], even in cases where humeral defects are considered non-engaging.

The recurrence rate observed in our study is consistent with previous findings. While a recent meta-analysis by Juan et al. [15] reported a recurrence rate of 7.3% following Bankart repair with remplissage, the shorter follow-up in that study (35.8 months) may explain the slightly lower failure rate compared to our cohort. Several studies have suggested that the risk of recurrence increases over time, especially in young, active populations where high-demand sports activities can expose the shoulder to repetitive trauma [4–6]. Our findings confirm that even with a longer follow-up period, Bankart repair combined with remplissage remains a durable solution with a relatively low recurrence rate.

When stratified by preoperative humeral head defect type, 19 patients (76%) had on-track lesions and 6 patients (24%) had off-track lesions. Among the three recurrences observed, two occurred in the on-track group (10.5%) and one in the off-track group (16.7%). The off-track recurrence required revision surgery with a bone-block procedure. Although our study did not include a control group treated with isolated Bankart repair, these findings suggest that the risk of recurrence persists even in first-time dislocators with on-track lesions, which supports the potential value of adding a remplissage procedure. Several recent studies [15, 23, 24] have shown that, in young athletic patients with on-track lesions, the combination of Bankart repair and remplissage significantly reduces recurrence rates compared to isolated Bankart repair, with reported rates ranging from 1.8 to 5% versus 11 to 30%, respectively. The main rationale for remplissage in these patients is biomechanical: Posterior tenodesis enhances humeral–glenoid coaptation and reduces the risk of recurrence during extreme shoulder loading. In a population of young, high-risk athletes, this preventive strategy may therefore help prolong shoulder stability and reduce the need for revision surgery in the mid- to long term.

Compared to the Latarjet procedure, which is widely used in high-risk or recurrent cases, our approach offers potential advantages in selected patients with first-time dislocations and on-track lesions. The Latarjet technique, while effective, is technically more demanding and carries risks such as graft non-union, neurovascular injury, excessive graft placement with intra-articular overhang, and limitations in external rotation [17]. In contrast, the Bankart + remplissage technique preserves the anatomy and avoids bone graft complications, with less surgical morbidity. This makes it an appealing option for young athletes in whom preservation of shoulder mobility and a low complication profile are particularly desirable.

Some studies have reported decreased shoulder mobility, particularly external rotation, following Bankart repair combined with remplissage due to the tenodesis of the infraspinatus tendon [25–27]. In our study, however, we did not observe this limitation. Patients exhibited satisfactory clinical scores, especially in subsection D (“global mobility”) of the Walch-Duplay score, which assesses external rotation among other factors. Our findings align with more recent studies that did not report significant loss of external rotation after remplissage [15, 23, 24]. Additionally, a biomechanical study by Argintar et al. demonstrated that the remplissage procedure does not significantly restrict shoulder mobility [28].

Thus, recent studies have shown that the recurrence rate of Bankart repairs increases over time, potentially reaching 25% in the presence of a non-engaging defect. Studies have demonstrated the superiority of the Bankart + remplissage procedure over isolated Bankart repairs, even for non-engaging lesions (Lin et domos [15, 16] Recent studies by Lin and Domos indicate significantly higher recurrence rates in the isolated Bankart group compared to the Bankart + remplissage group (1.8% vs. 11% and 5% vs. 30%, respectively) [29].

One of the key strengths of our study is its focus on first-time dislocations. The management of initial anterior shoulder dislocations in young athletes remains a subject of debate. While some authors advocate for early surgical stabilization to reduce the risk of recurrence and long-term functional impairment [4, 7, 11], others argue that conservative treatment may be sufficient in selected cases. However, the high recurrence rates reported in non-surgical cohorts ranging from 50 to 90% in young athletes [1–3] support the rationale for early surgical intervention. Our study suggests that combining Bankart repair with remplissage may offer an additional advantage by further reducing recurrence, even in cases traditionally considered at lower risk due to non-engaging defects.

The functional outcomes in our study were excellent, with a mean Walch-Duplay score of 89.5, a mean WOSI score of 10.95, and a mean Quick-DASH score of 14.30. These results align with existing literature, where remplissage has been associated with significant improvements in functional scores and patient-reported outcomes [19, 21, 22]. Villarreal-Espinosa et al. reported a substantial improvement in the Rowe score (92.2 postoperatively vs. 43.9 preoperatively) after Bankart repair combined with remplissage, further supporting the efficacy of this approach in restoring shoulder stability [15].

In our study, the return-to-sport rate was also highly favorable, with 84% of patients resuming their pre-injury level of activity or higher. This is particularly important for competitive and recreational athletes, as persistent shoulder instability can significantly impact performance and participation.

However, the study has limitations, including a small sample size (25 patients), which limits statistical power and prevents a comprehensive analysis of risk factors for failure. Further research with larger sample sizes or additional variables may be required to conclusively identify significant risk factors. The absence of a control group (comparison with isolated Bankart repair) means we cannot conclusively determine the specific benefit of remplissage. Additionally, the retrospective nature of the study introduces a potential recall bias, though this is likely minimal for the primary outcome, given its significant impact on patients. A prospective, comparative study with a larger sample size would help to confirm these results.

## Conclusion

With a 5-year failure rate of 12%, capsulolabral reconstruction with humeral remplissage may be considered as a possible first-line treatment for young patients with a first episode of LGHA, especially if they are athletes. A prospective, comparative study would be valuable to confirm these results.

## Data Availability

No datasets were generated or analysed during the current study.

## References

[1] Lill H, Verheyden P, Korner J, Hepp P, Josten C (1998) Konservative Behandlung nach traumatischer Schultererstluxation? Chirurg 69(11):1230–1237 9864635 10.1007/s001040050561

[2] Robinson CM, Howes J, Chb M, Murdoch H, Chb M, Will E, et al Functional Outcome and Risk of Recurrent Instability After Primary Traumatic Anterior Shoulder Dislocation in Young Patients. 11 10.2106/JBJS.E.01327 17079387

[3] Hovelius L, Augustini BG, Fredin H, Johansson O, Norlin R, Thorling J (1996) Primary anterior dislocation of the shoulder in young patients. A ten-year prospective study. J Bone Joint Surg 78(11):1677–1684 8934481 10.2106/00004623-199611000-00006

[4] Jakobsen BW, Johannsen HV, Suder P, Søjbjerg JO (2007) Primary repair versus conservative treatment of first-time traumatic anterior dislocation of the shoulder: a randomized study with 10-year follow-up. Arthrosc J Arthrosc Relat Surg févr 23(2):118–123 10.1016/j.arthro.2006.11.004 17276217

[5] Arciero RA, Wheeler JH, Ryan JB, McBride JT (1994) Arthroscopic Bankart repair versus nonoperative treatment for acute, initial anterior shoulder dislocations. Am J Sports Med 22(5):589–594 7810780 10.1177/036354659402200504

[6] Bottoni CR, Wilckens JH, DeBerardino TM, D’Alleyrand JCG, Rooney RC, Harpstrite JK et al (2002) A prospective, randomized evaluation of arthroscopic stabilization versus nonoperative treatment in patients with acute, traumatic, first-time shoulder dislocations. Am J Sports Med juill 30(4):576–580 10.1177/03635465020300041801 12130413

[7] Kirkley A, Werstine R, Ratjek A, Griffin S (2005) Prospective randomized clinical trial comparing the effectiveness of immediate arthroscopic stabilization versus immobilization and rehabilitation in first traumatic anterior dislocations of the shoulder: Long-term evaluation. Arthrosc J Arthrosc Relat Surg janv 21(1):55–63 10.1016/j.arthro.2004.09.018 15650667

[8] Uhring J, Rey PB, Rochet S, Obert L (2014) Intérêt d’une stabilisation arthroscopique en urgence lors du premier épisode de luxation gléno-humérale antérieure chez le jeune sportif. Rev Chir Orthop Traumatol déc 100(8):S375–S383

[9] De Carli A, Vadalà AP, Lanzetti R, Lupariello D, Gaj E, Ottaviani G et al (2019) Early surgical treatment of first-time anterior glenohumeral dislocation in a young, active population is superior to conservative management at long-term follow-up. Int Orthop 43(12):2799–2805 31392495 10.1007/s00264-019-04382-2

[10] Gigis I, Heikenfeld R, Kapinas A, Listringhaus R, Godolias G (2014) Arthroscopic versus conservative treatment of first anterior dislocation of the shoulder in adolescents. J Pediatr Orthop juin 34(4):421–425 10.1097/BPO.0000000000000108 24172677

[11] Pougès C, Hardy A, Vervoort T, Amouyel T, Duriez P, Lalanne C et al (2021) Arthroscopic Bankart Repair Versus Immobilization for First Episode of Anterior Shoulder Dislocation Before the Age of 25: A Randomized Controlled Trial. Am J Sports Med 49(5):1166–1174 33705240 10.1177/0363546521996381

[12] Chapus V, Rochcongar G, Pineau V, Salle de Chou É, Hulet C (2015) Ten-year follow-up of acute arthroscopic Bankart repair for initial anterior shoulder dislocation in young patients. Orthop Traumatol Surg Res. 101(8):889 26563924 10.1016/j.otsr.2015.09.029

[13] Murphy AI, Hurley ET, Hurley DJ, Pauzenberger L, Mullett H (2019) Long-term outcomes of the arthroscopic Bankart repair: a systematic review of studies at 10-year follow-up. J Shoulder Elbow Surg 28(11):2084–2089 31311748 10.1016/j.jse.2019.04.057

[14] Burkhart SS, De Beer JF (2000) Traumatic glenohumeral bone defects and their relationship to failure of arthroscopic Bankart repairs. Arthrosc: J Arthrosc Relat Surg 16(7):677–694 10.1053/jars.2000.17715 11027751

[15] Villarreal-Espinosa JB, Saad Berreta R, Cotter E, Rafael Garcia J, Gonzalez Ayala S, Khan ZA, et al. (2024) Lower Range of Recurrent Instability Rates Following Bankart Repair and Remplissage Compared to Isolated Bankart Repair in Patients With “Nonengaging/On-Track” Hill-Sachs Lesions and < 20% Glenoid Bone Loss. Arthrosc J Arthrosc Relat Surg. S0749806324003426. 10.1016/j.arthro.2024.04.036 38735408

[16] Villarreal-Espinosa JB, Saad Berreta R, Cotter E, Rafael Garcia J, Gonzalez Ayala S, Khan ZA et al (2025) Lower Range of Recurrent Instability Rates Following Bankart Repair and Remplissage Compared to Isolated Bankart Repair in Patients With “Nonengaging/On-Track” Hill-Sachs Lesions and <20% Glenoid Bone Loss. Arthrosc J Arthrosc Relat Surg avr 41(4):1085–1095 10.1016/j.arthro.2024.04.036 38735408

[17] Griesser MJ, Harris JD, McCoy BW, Hussain WM, Jones MH, Bishop JY et al (2013) Complications and re-operations after Bristow-Latarjet shoulder stabilization: a systematic review. J Shoulder Elbow Surg févr 22(2):286–292 10.1016/j.jse.2012.09.009 23352473

[18] Di Giacomo G, Di Giacomo G, Itoi E, Burkhart SS (2014) Evolving concept of bipolar bone loss and the Hill-Sachs lesion: from « engaging/non-engaging » lesion to « on-track/off-track » lesion. Arthroscopy 30(1):90–98. 10.1016/j.arthro.2013.10.004 24384275 10.1016/j.arthro.2013.10.004

[19] Khiami F, Sariali E, Rosenheim M, Hardy P (2012) Anterior shoulder instability arthroscopic treatment outcomes measures: The WOSI correlates with the Walch-Duplay score. Orthop Traumatol Surg Res 98(1):48–53 22204794 10.1016/j.otsr.2011.09.013

[20] Beaton DE, Wright JG, Katz JN (2005) Development of the QuickDASH: comparison of three item-reduction approaches. 10.2106/JBJS.D.02060 15866967

[21] Harvie P, Pollard TCB, Chennagiri RJ, Carr AJ (2005) The use of outcome scores in surgery of the shoulder. J Bone Joint Surg Br 87:151–4 15736732 10.1302/0301-620x.87b2.15305

[22] Wolf EM, Arianjam A (2014) Hill-Sachs remplissage, an arthroscopic solution for the engaging Hill-Sachs lesion: 2- to 10-year follow-up and incidence of recurrence. J Shoulder Elbow Surg juin 23(6):814–820 10.1016/j.jse.2013.09.009 24295834

[23] Lin A, Barrow AE, Charles S, Shannon M, Fox MA, Herman ZJ et al (2023) Remplissage reduces recurrent instability in high-risk patients with on-track Hill-Sachs lesions. J Shoulder Elbow Surg juin 32(6):S99-105 10.1016/j.jse.2023.02.011 36828289

[24] Domos P, Ascione F, Wallace AL (2019) Arthroscopic bankart repair with remplissage for non-engaging Hill-Sachs lesion in professional collision athletes. Shoulder Elbow 11(1):17–25 30719094 10.1177/1758573217728414 PMC6348582

[25] Nourissat G, Kilinc AS, Werther JR, Doursounian L (2011) A prospective, comparative, radiological, and clinical study of the influence of the “Remplissage” procedure on shoulder range of motion after stabilization by arthroscopic Bankart repair. Am J Sports Med 39(10):2147–2152 21816983 10.1177/0363546511416315

[26] Deutsch AA, Kroll DG (2008) Decreased range of motion following arthroscopic remplissage. Orthopedics. 31(5):1–3 10.3928/01477447-20080501-07 19292311

[27] Zhu YM, Lu Y, Zhang J, Shen JW, Jiang CY (2011) Arthroscopic bankart repair combined with remplissage technique for the treatment of anterior shoulder instability with engaging Hill-Sachs lesion: a report of 49 cases with a minimum 2-year follow-up. Am J Sports Med août 39(8):1640–1648 10.1177/0363546511400018 21505080

[28] Argintar E, Heckmann N, Wang L, Tibone JE, Lee TQ (2016) The biomechanical effect of shoulder remplissage combined with Bankart repair for the treatment of engaging Hill-Sachs lesions. Knee Surg Sports Traumatol Arthrosc févr 24(2):585–592 10.1007/s00167-014-3092-4 24912574

[29] Lin KM, James EW, Spitzer E, Fabricant PD (2018) Pediatric and adolescent anterior shoulder instability: clinical management of first-time dislocators. Curr Opin Pediatr févr 30(1):49–56 10.1097/MOP.0000000000000566 29135565

